# App Features for Type 1 Diabetes Support and Patient Empowerment: Systematic Literature Review and Benchmark Comparison

**DOI:** 10.2196/12237

**Published:** 2018-11-21

**Authors:** Antonio Martinez-Millana, Elena Jarones, Carlos Fernandez-Llatas, Gunnar Hartvigsen, Vicente Traver

**Affiliations:** 1 ITACA Universitat Politècnica de València Valencia Spain; 2 Department of Computer Science University of Tromsø Tromsø Norway

**Keywords:** mHealth, type 1 diabetes mellitus, patient empowerment, apps, diabetes self-management

## Abstract

**Background:**

Research in type 1 diabetes management has increased exponentially since the irruption of mobile health apps for its remote and self-management. Despite this fact, the features affect in the disease management and patient empowerment are adopted by app makers and provided to the general population remain unexplored.

**Objective:**

To study the gap between literature and available apps for type 1 diabetes self-management and patient empowerment and to discover the features that an ideal app should provide to people with diabetes.

**Methods:**

The methodology comprises systematic reviews in the scientific literature and app marketplaces. We included articles describing interventions that demonstrated an effect on diabetes management with particular clinical endpoints through the use of mobile technologies. The features of these apps were gathered in a taxonomy of what an ideal app should look like to then assess which of these features are available in the market.

**Results:**

The literature search resulted in 231 matches. Of these, 55 met the inclusion criteria. A taxonomy featuring 3 levels of characteristics was designed based on 5 papers which were selected for the synthesis. Level 1 includes 10 general features (Personalization, Family support, Agenda, Data record, Insulin bolus calculator, Data management, Interaction, Tips and support, Reminders, and Rewards) Level 2 and Level 3 included features providing a descriptive detail of Level 1 features. Eighty apps matching the inclusion criteria were analyzed. None of the assessed apps fulfilled the features of the taxonomy of an ideal app. Personalization (70/80, 87.5%) and Data record (64/80, 80.0%) were the 2 top prevalent features, whereas Agenda (5/80, 6.3%) and Rewards (3/80, 3.8%) where the less predominant. The operating system was not associated with the number of features (*P*=.42, F=.81) nor the type of feature (*P*=.20, χ^2^=11.7). Apps were classified according to the number of level 1 features and sorted into quartiles. First quartile apps had a regular distribution of the ten features in the taxonomy whereas the other 3 quartiles had an irregular distribution.

**Conclusions:**

There are significant gaps between research and the market in mobile health for type 1 diabetes management. While the literature focuses on aspects related to gamification, rewarding, and social communities, the available apps are focused on disease management aspects such as data record and appointments. Personalized and tailored empowerment features should be included in commercial apps for large-scale assessment of potential in the self-management of the disease.

## Introduction

Diabetes mellitus is a metabolic syndrome, which comprises an impaired insulin production and action [1]. Type 1 diabetes mellitus (T1DM) prevalence is rapidly rising throughout the world [2]. Supporting T1DM patients is a major health care challenge, as it involves many aspects of daily routine activities (eg, food intake, physical activity, motivation) and specific knowledge of disease mechanisms (eg, blood glucose regulation, insulin intake) [3,4].

Despite the promise of mobile health (mHealth) in the specific field of diabetes [5,6] and the explosion of diabetes-related apps in markets, T1DM management is yet undertaken on a routine care basis, in which glycemic levels (blood glucose and hemoglobin A_1c_ (HbA_1c_) among others) and other health-related outcomes are supervised by general practitioners, endocrinologists, and nurses. This type of care has shown limited effects on empowering patients to control blood sugar levels [7].

In their review from 2011, Chomutare et al [8] compared the recommendations from evidence-based guidelines and the features of mobile apps. The evaluation of features was analyzed for 6 types of functionalities: (1) self-monitoring, (2) education, (3) alerts, (4) reminders, (5) social media, and (6) personal health records synchronization. The authors concluded that apps did not include education structural elements from the empowerment of patients other than including functionalities for routine management (record of lifestyle and measurements). The lack of core recommendations compromised the effectiveness of mobile health in diabetes clinical management [9].

Since then, several studies have been conducted to test the effectiveness of apps in reduced samples of patients. In such studies, researchers evaluate apps with different approaches and patient groups, yielding conflicting conclusions due to the different methodology of the interventions [10].

This study aimed to assess whether app manufacturers adopted the findings from mHealth evidence-based interventions in diabetes. The rationale is to mind the gap between research and the market to identify the features that are not available in commercial apps. The methodology comprises 2 systematic reviews using the PRISMA methodology, one in the scientific literature (without meta-analysis) and the other in app marketplaces. The systematic review of scientific literature is focused on interventions with apps on patients with T1DM into randomized controlled trials (RCTs). In the first stage, we built the schema of what could be an ideal diabetes app (including all the features which have shown a positive effect on diabetes management) and to then analyze the characteristics of available apps and their distance to the ideal app.

## Methods

### Selection Criteria

The primary objective of this study was to review scientific literature using the PRISMA methodology to enumerate evidence-based features that have demonstrated a positive effect in the management of T1DM in RCTs. Inclusion criteria were defined as (1) a mHealth intervention on T1DM patients using an app for remote and self-management of the disease and (2) the intervention was performed in an RCT and reports on clinical outcomes (HbA_1c_, in-range time or self-monitoring blood glucose). Exclusion criteria included (1) gray literature, (2) studies not reporting RCTs on T1DM (eg, type 2 diabetes, gestational diabetes), and (3) studies not using an app (eg, text messages, manual notes) or not describing the app’s functionality. The secondary objective was to compare the evidence-based features with the characteristics of the available apps.

### Recruitment Strategy

The source of the literature review were online journal databases and indexers (PubMed, Medline, Google Scholar, and Cochrane Trials). We searched a combination of keywords including type 1 diabetes mellitus, mobile health, RCTs and self-management. The complete search strategy, combination of keywords in the queries and results for PubMed are described in Multimedia Appendix 1. The search included all the parts of the manuscript (title, abstract, keywords, and text). The review was conducted in July 2018, and we focused our review on recently published papers (January 2012-July 2018). Only publications in English were considered for inclusion.

Subsequently, the approach for recruiting apps was twofold. First, a web search was conducted by using keywords of 3 different groups: (1) “diabetes mellitus 1 AND apps AND (android OR iPhone) AND self-management,” (2) “diabetes mellitus,” and (3) “diabetes apps AND self-management.” A second search was conducted in Google Play and the App Store to recruit a greater number of apps. In this case, we introduced 2 keywords: “diabetes AND management.” After collecting all the available apps, the screening and selection were done in the same way as with the publications (using a PRISMA flow diagram). Subsequently, the selected apps were downloaded and tested to know which of the characteristics obtained with the systematic literature review were available in the apps.

Data from the literature review and the apps assessment was extracted by 2 of the authors (AMM and EJP) using a structured data form. For the literature review, we extracted data related to the study (year of publication, sample size, the age of participants, methodology, intervention, clinical endpoints, features, usability, and satisfaction). Studies were assessed using the Cochrane Collaboration’s tool to assess the risk of bias of included (selection, reporting, performance, and attrition) [11]. For the app review, we extracted the type of features each app offered, the operative system, language, icon, and link.

### Statistical Analysis

A descriptive analysis of the features in the apps was done before association analytics. Association of the type of features and the type of operative system was evaluated with a two-way analysis of variance, in which we assessed the *P* value and the F statistic. Association of the number of features and the operative system was assessed using Kruskal-Wallis tests at 95% CI, in which we calculated the *P* value and the chi-square value. A value of *P*<.05 was considered as significant The Feature Factor (FF) was defined as the polynomial formula (Equation 1) FF= N_L1_^3^+N_L2_^2^+N_L3_ where N_Lx_ is the number of features in level 1, level 2, and level 3 respectively. We used MATLAB 2017Ra framework under Academic License to conduct the statistical analysis.

## Results

### Systematic Literature Review of Features

**Figure 1:**
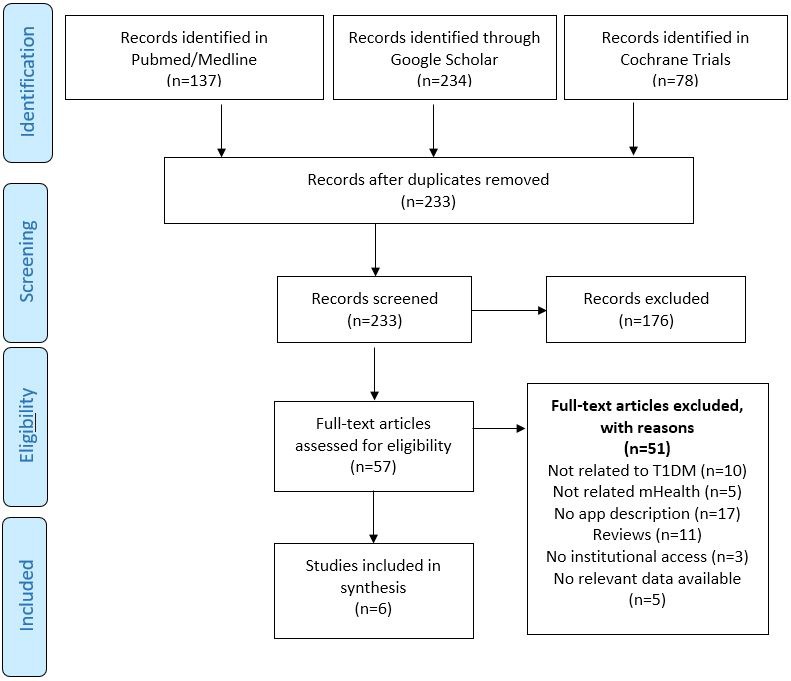
Selection of the literature for evidence-based features of mobile apps for type 1 diabetes management and empowerment.

Figure 1 shows the cascade flow of search process for the literature review. A total of 449 scientific publications were found in the selected search engines with the chosen keywords and the date constraints. After excluding 216 duplicated entries, we obtained a total of 233 original publications. In the second screening of these 233 articles, 176 were not related to the objective of this study and were eliminated. A big part of the remaining 51 articles was not matching the inclusion criteria. Ten of the 51 (19.6%) did not refer to T1DM, 5/51 (9.8%) were not related to mHealth, 11/51 (21.6%) were reviews, 17/51 (33.3%) were not in the scope of our study, 3/51 (5.9%) did not allow full-text publication accessibility, and 5/51 (9.8%) did not provide relevant data for the synthesis. Finally, once read and analyzed, 6/57 (11.8%) scientific publications were included for the synthesis [12-17].

These 6 studies reported on the results evaluating the benefits of apps in the management of T1DM (Table 1). These studies consisted of 266 participants (as we only considered intervention groups) in RCTs and lasted from 3 to 12 months. Only studies with a clear clinical endpoint were considered (eg, HbA_1c_ improvement, self-monitoring blood glucose (SMBG) daily rate).

**Table 1:** Characteristics of the studies evaluating mobile apps for type 1 diabetes mellitus management and empowerment.

Characteristics	Castensøe-Seidenfaden [12]	Cafazzo [13]	Goyal [14]	Kirwan [15]	Clemens [16]	Ryan [17]
Publication year	2018	2012	2017	2013	2017	2017
Intervention, n	76	20	46	25	81	18
Age (years), mean (SD)	17.6 (2.6)	14.9 (1.3)	14.1 (1.7)	35.9 (10.6)	14.0 (10.4-15.9)^a^	40 (13.9)
Time^b^ (years), mean (SD)	8.0 (4.5)	NS^c^	7.1 (3.2)	19.7 (9.6)	4.9 (2.7-7.5)^a^	27.3 (14.9)
Duration (months)	12	3	12	6	3.6	4
Intervention type	Usual care App	App	Usual care App	App Feedback	Retrospective analysis	Usual care App
App name	Young with Diabetes	Bant	Bant	Glucose Buddy	NS	NS
HbA_1c_^d^ outcome	No significant change	No significant change	Decrease by 0.58% (*P*=.02)	Decrease in mean (SD) from 9.08% (1.18%) to 7.8% (0.75%)	No significant change	Decrease in median (9.1% to 7.8%)
SMBG^e^ outcome	—	Increased mean daily frequency (2.4 to 3.6, *P*=.006, n=12)	—	No significant change	Increased 2.3 times	—
App perceived usefulness^f^	Chat Room (among young people)	Reminders, blood glucose regulation, insulin and food regulation, emergency readiness, exercise	Trending feature, logbook, and home menu (statistics)	NA^g^; texting extensively used	Data synchronization	Bolus calculator and glucose control. Badges used by 17%
User satisfaction	>80% would recommend	88% would continue to use	76% “satisfied/very satisfied” 96% would continue using app	NA	NA	NA

^a^Median and interquartile range.

^b^Since diagnosis.

^c^NS: not specified.

^d^HbA_1c_: hemoglobin A_1c_.

^e^SMBG: self-monitoring blood glucose.

^f^Either a 5-point or 10-point Likert scale was used to score.

^g^NA: not assessed.

Overall, the included trials adequately achieved a low level of risk of bias (Table 2). Three of the studies did not report on random sequence generation, and 2 did not adequately report concealing the allocation sequence to determine if the group could have been foreseen. Only 2 trials blinded the outcome assessment. Besides, all trials except 1 adequately described data completeness (including exclusion and drops out) and did not perform selective outcome description.

The majority of these studies assessed the features of the app which had a higher perceived usefulness or a good adoption among study subjects. Castensøe-Seidenfaden et al [12] found the Chat Room as the most rated feature, a virtual space for user communication in which users posted comments about alcohol and sex. Cafazzo et al [13] report the user-centered design and evaluation in 12 subjects of the Bant app; a mobile app that’s focused on the simple and automated transfer of glucometer readings, a social community and rewards for healthy behavior adherence (average 8 rewards per user in a three-month trial). An updated version of the Bant app was tested by Goyal et al [14] in a 12-months trial involving 46 subjects, pointing out the trends, logbook, and homepage as the preferred features of the app. Kirwan et al [15] examined the effectiveness of Glucose Buddy, a free app combined with texting from a certified diabetes educator in 25 subjects.

**Table 2:** Cochrane Collaboration’s tool risk-of-bias assessment for the clinical outcomes (hemoglobin A_1c_ and self-monitoring blood glucose changes) of the included study papers.

	Castensøe-Seidenfaden [12]	Cafazzo [13]	Goyal [14]	Kirwan [15]	Clemens [16]	Ryan [17]
Random sequence generation	Low	High	Low	Low	High	High
Allocation concealment	Low	High	Low	Low	High	Low
Blinding of participants and personnel	N/A^a^	N/A	N/A	N/A	N/A	N/A
Blinding of outcome assessment	Low	High	High	Low	High	Unclear
Incomplete outcome data	Low	Low	Low	High	Low	Low
Selective reporting	Low	Low	Low	High	Low	Low
Other bias	Low	Unclear	Low	Unclear	Unclear	Low

^a^N/A: not applicable (both patient and doctors know the group they are allocated).

Clemens and Staggs [16] reported a retrospective study on 81 youth T1DM patients who used a mobile app connected to a glucose meter, showing that data synchronizations were associated with an increased rate of SMBG but not with HbA_1c_ or mean glucose values. Beyond data synchronization, no more app features were provided by authors in this paper. Finally, Ryan et al [17] developed and evaluated Intelligent Diabetes Management, an app for insulin bolus calculation and an electronic diabetes diary in clinical visits. The app included a “badge” feature for motivational accolades based on the reported measurements. The 18 subjects, who completed the study, rated the insulin calculator with 8 in a 10-point Likert scale and only 17% of subjects used the “badges” feature regularly.

Three of these studies evaluated the user satisfaction in terms of willingness to use the app after the trial and willingness to recommend its use to peers. Each showed a high percentage of subjects willing to continue using the app and willing to recommend the app to peers [12-14]. In our study, we analyzed the features available in the mobile apps (rated or not) and gathered in a hierarchical map of functionalities.

### Features Taxonomy of an Ideal App

**Figure 2:**
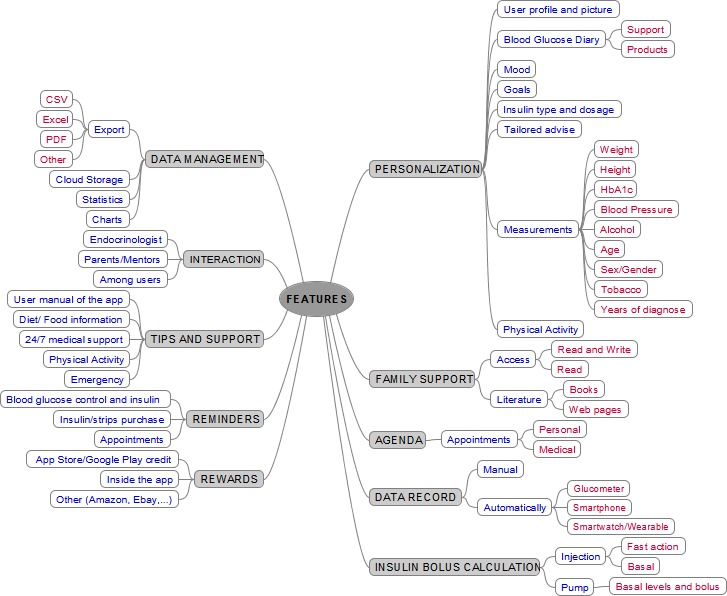
Taxonomy of the features of an ideal app according to the evidence-based effectiveness of mobile health in diabetes support and empowerment.

The qualitative study of the apps used in the studies allowed us to extract the characteristics of the apps that users rated as key and researchers considered of value. These features were sorted into functional areas beyond the traditional 4 clinical areas of diabetes management taxonomy (glycemic control, carbohydrate intake, insulin and exercise) [18]. The new proposed taxonomy (Figure 2) had 3 hierarchical levels, the first of which had 10 areas: (1) Personalization, (2) Family support, (3) Agenda, (4) Data record, (5) Insulin bolus calculation, (6) Data management, (7) Interaction, (8) Tips and support, (9) Reminders, and (10) Rewards. In this area, we included all the functions and settings that users can adjust to customize the app interface, the behavior, and calculations (if any). The basic feature was building a user profile for the social community of app users, with the capability of adding a picture or avatar. The second feature in Personalization was the possibility of configuring the blood glucose diary (or record), adjusting personalized thresholds for supporting the patient, and product recommendation. The third feature was the configuration of the user mood. The fourth feature was the self-adjustment of goals of any type (glucose goals, carbs intake, weight loss). The fifth feature was setting up the type of insulin, the brand, and the intake method. The sixth feature was configuring tailored advices (eg, topic selection, when to show the advice). The seventh feature was the personalization of the measurements reporting, instead of having a default form for data introduction, for all the relevant variables (Figure 2). The last feature of personalization was the customization of physical activity reporting.

Family support was a feature in which relatives can perform a follow-up of the data introduced by the patient in which we have identified read access and read-and-write access. This feature also contains literature support for relatives in the management of T1DM through web links and books. The Agenda feature was a common factor in the apps analyzed in the review, and it was mainly devoted for scheduling medical and personal appointments. Another recurrent feature was the storage of measurements and reports related to food intake, insulin intake, and physical activity, which are under the Data record feature. In this feature, we made a distinction between the apps that had manual data entry (eg, forms, pictures, voice recognition, avatar) and the automatic entry of data using wireless sensors, mobile phone sensors (such as an accelerometer for physical activity) and smartwatches and wearables. The last feature (right side in Figure 2) was the Insulin bolus calculator, which provided calculations for exogenous insulin injection for fast action (bolus) or slow action (basal), and for the insulin pump device (bolus and basal).

The Data management feature involved the capability of the app of exporting, storing, and analyzing the data collected in the app. In the review, one of the top-rated features was the graphical representation of measurements and the calculation of statistics based on these measurements. The second feature in the right side of Figure 2 refers to the ability of the app to interact with other subjects beyond the T1DM user (clinicians, parents, and other app users). This interaction was generic and involves social communities, participation in forums, and data sharing. All the analyzed apps had a feature for Tips and support, which involved a user manual of the app. Some of them also included generic information related to diet and physical activity. Only 1 had online 24/7 medical support and emergency management. The Reminders feature enabled users to set up alerts for measuring and recording blood glucose tests, insulin intakes, insulin and glucometer strip purchasing, and attending medical or personal appointments scheduled in the Agenda. Finally, the Reward feature contained the motivation mechanisms that the app implements for control and therapy adherence. Rewarding strategies in the review were related for providing credit in virtual markets, premium access to the app (rewards for enhanced functionalities) and other types of credit for online stores (Amazon, eBay vouchers).

### Systematic Review of Commercial Diabetes Management Apps

**Figure 3:**
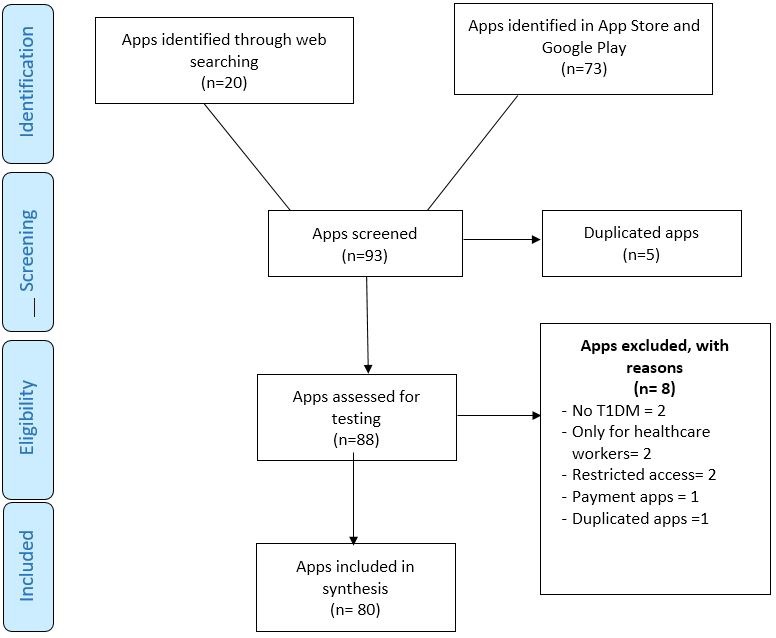
Selection of the available apps for type 1 diabetes management and empowerment.

**Figure 4:**
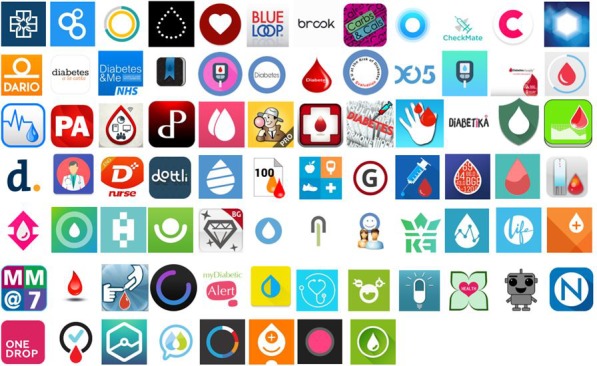
Apps included in the analysis. From top-down left-right: Balansio, Bant, BeatO, Beyond type 1 diabetes, Blood glucose tracker, BlueLoop, Brook, Carbs & Cals, Center health, Checkmate diabetes, Chron, Contour diabetes, Dario, Diabetes a la carta, Diabetes & Me, Diabetes connect, Diabetes diary, Diabetes diet and management, Diabetes digest, Diabetes evaluation, Diabetes experience day, Diabetes ID, Diabetes insight, Diabetes kit blood, Diabetes metrics, Diabetes PA, Diabetes pal, Diabetes parent, Diabetes passport, Diabetes pilot pro, Diabetes plus, Diabetes treatment, Diabetes vue, Diabetika, Diaguard, Diario de sangre, Diasend, DMI from zero to hero, Dnurse, Dottli, Dr. Diabetes, Easy diabetes, Glooko, Glucool diabetes, Glucosa compañero, Glucose buddy, Glucose wiz, Uright, Glucosio, GluQUO, Health2sync, Helparound, iFora, Inrange, Insulclock, Kids and teens diabetes, Kingfit, La diabetes M, Life in control, MedM diabetes, Meet me, Mi glucemia, Monitor de glucosa, Mumoactive, My diabetic alert, Nagbot, Neptun, One drop, Ontrack diabetes, PredictBGL, Social diabetes, SOS diabetes, Sugar sense, Sugarmate, Track3lite.

We evaluated the available apps through an online search, the App Store (Apple) and Google Play (Android) for diabetes management and support. Following the PRISMA methodology approach (Figure 3), we obtained 93 single apps devoted to T1DM (20/93 (21.5%) from the online search and 73/93 (78.5%) from the previously mentioned app markets). Five of the 93 apps (5.4%) were excluded because the links for downloading the software were unavailable. Of the remainder, 8/87 (91.9%) apps were excluded from the detailed analysis: 2/87 (2.3%) because they were not tailored to T1DM, 2/87 (2.3%) because they focused on healthcare professionals, 2/87 (2.3%) because the access was secured with a password (corporate use), 1/87 (1.1%) because it was not free and 1 (1.1%) because it was a copy of another app with different name and icon. Finally, 80 apps (Figure 4) were assessed to discover the extent to which these apps fulfilled the feature schema of an ideal app taxonomy.

Android and iOS apps were tested during a month since many apps needed control over a longer period to provide data (graphics, statistics) to the user. A mHealth expert analyzed the apps and collected all the features which matched the taxonomy of the ideal diabetes management app. Features were flagged with a Boolean value depending on whether the app provided the analyzed feature. Occasionally, the mHealth expert was provided with clarification to ensure that the analysis was more precise.

### The Gap Between Literature and Apps in Diabetes Management

None from the 80 analyzed apps fulfilled the criteria of the taxonomy of an ideal app. Only BlueLoop had 9 out of the 10 ideal features in level 1. Table 3 describes the distribution of features amongst the analyzed apps. Sixteen of the 80 (19.7%) apps were only for Android, 15/80 (18.5%) only for iOS, and 50/80 (62.7%) for both operative systems. The mean for the number of level 1 features in the analyzed apps was 4.6 (SD 1.7) with interquartile range 3-6 and range 2-9. Based on the taxonomy of an ideal app, the predominant category of features was Personalization (70/80, 87.5%), followed by Data record (64/80, 80.0%), Tips and support and Data management (60/80, 75.0%). Reminders were featured in less than a half (33/80, 40.7%), Family support in 37.5% (30/80), and Agenda in 36.3% (29/80). Less than a third (23/80, 28.7%) featured Interaction and 25.0% (20/80) had Insulin bolus calculation. The least predominant feature was Rewards (3/80, 3.8%). The number of level 1 features was not associated with the operative system (*P*=.42, F=.81). In the same way, the level 1 type of feature was not associated with the operative system (*P*=.20, X^2^_29_=11.7).

**Table 3:** Feature frequency in the reviewed diabetes management apps for the 3 hierarchy levels.

Level hierarchy			Apps containing the feature, n (%)
**Personalization (n=70)**			70 (100.0)
	User profile and picture		40 (57.1)
	Blood glucose diary		64 (91.4)
	Mood		14 (20.0)
	Goals		33 (47.1)
	Insulin type and dosage		21 (30.0)
	Tailored advice		8 (11.4)
	**Measurements**		24 (34.3)
		Weight	24 (34.3)
		Height	14 (20.0)
		Hemoglobin A_1c_	17 (24.3)
		Blood pressure	15 (21.4)
		Alcohol	1 (0.1)
		Age	6 (8.6)
		Gender	14 (0.2)
		Tobacco	1 (0.1)
		Years with type 1 diabetes mellitus	12 (17.1)
	Physical activity		35 (50.0)
**Family support (n=30)**			30 (100.0)
	**Access**		30 (100.0)
		Read and write	8 (26.7)
		Read	22 (73.3)
	**Literature**		7 (23.3)
		Books	12 (40.0)
		Web pages	7 (23.3)
**Agenda (n=29)**			29 (100.0)
	Appointments		29 (100.0)
**Data record (n=64)**			64 (100.0)
	Manual		62 (96.9)
	**Automatically**		30 (46.9)
		Glucometer	27 (42.2)
		Mobile phone	17 (26.6)
		Smartwatch/wearable	1 (1.6)
**Insulin bolus calculation (n=20)**			20 (100.0)
	**Injection**		17 (85.0)
		Bolus	9 (45.0)
	**Pump**		7 (35.0)
		Basal	9 (45.0)
**Data management (n=60)**			60 (100.0)
	**Export**		34 (56.7)
		Comma-separated values	16 (26.7)
		Excel	6 (10.0)
		PDF	10 (16.7)
		Other	20 (33.3)
	Cloud storage		28 (46.7)
	Statistics		18 (30.0)
	Charts		51 (85.0)
**Interaction (n=23)**			23 (100.0)
	Endocrinologists		16 (69.6)
	Parents/mentors		14 (60.9)
	Among users		9 (39.1)
**Tips and support (n=60)**			60 (100.0)
	User manual of the app		46 (76.7)
	Diet/food information		28 (46.7)
	24/7 medical support		7 (11.7)
	Emergency		11 (18.3)
**Reminders (n=33)**			33 (100.0)
	Blood glucose control and insulin		33 (100.0)
	Insulin/strips purchase		28 (84.8)
	Appointments		29 (87.9)
**Rewards (n=3)**			3 (100.0)
	App Store/Google Play credit		0 (0.0)
	Inside the app		3 (100.0)
	Other (Amazon, eBay, etc.)		1 (33.3)

Level 2 features in Personalization were dominated by the Blood glucose diary (64/70, 91.4%), whereas Tailored advice was only present in 11.4% (8/70). If we look into level 3 Measurements in Personalization, only 0.1% (1/70) of the apps included Tobacco and Alcohol, and 17.1% (12/70) included a field for Years of diagnosis. With regards to Family support, 100.0% (30/30) featured Read access, and 26.7% (8/30) included Writing access to relatives.

Fifty-one of 60 apps (85.0%) featuring Data management had the possibility of drawing charts with the stored measurements, and more than a half (34/60, 56.7%), offered the possibility of data exportation in several formats. Nearly a half (28/60, 46.7%) offered the possibility of storing data in the Cloud, and only 30.0% (18/60) had the option of calculating statistics.

Level 2 features of Tips and support showed that not all the apps have a user manual (46/60, 76.7%), less than a half (28/60, 46.7%) provided Diet/food information, only 11.67% (7/60) had 24/7 Medical support specific for diabetes, and 18.3% (11/60) had Emergency support.

All the Reminders (33/33, 100%) were for blood glucose and insulin schedule. A majority were for purchasing fungibles (28/33, 84.8%) and Appointments (29/33, 87.9%). Three of 3 (100%) provided Rewards inside the app, and only 1/3 (33.3%) gave Rewards in online markets.

**Figure 5:**
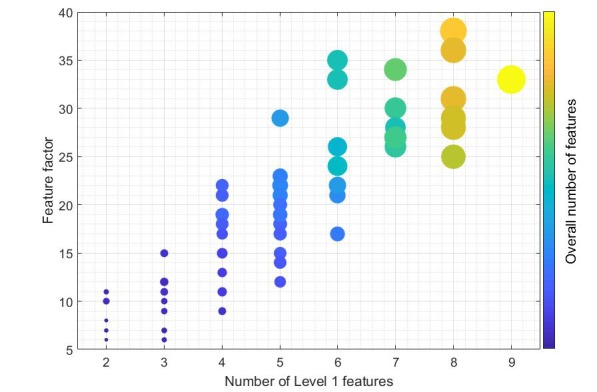
The relationship among the number of level 1 features and the Feature Factor.

Figure 5 shows the relationship between level 1 features and the FF as defined in Equation 1. The size and color of the point indicates the level of feature completeness according to the ideal app taxonomy. The yellow point refers to the app which had 9 of the 10 level 1 features, which is surpassed by 2 apps with 8 level 1 features, but which also implement more level 2 features.

**Figure 6:**
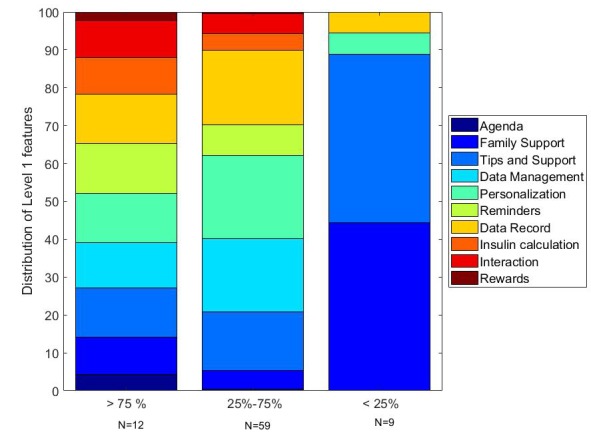
Distribution of level 1 features classified according to the number of level 1 features in the apps divided into quartiles.

Apps were classified according to the number of level 1 features they offered. Figure 6 shows the distribution of these features in a quartile classification of the number of level 1 features variable. First quartile (>75% in Figure 6) shows a regular distribution of the features, except to Agenda and Rewards. Second and third quartile apps (25%-75% in Figure 6) have a heterogeneous distribution of features, with an increased presence of Reminders, Personalization and Data management, and a lower presence of Insulin calculation, Agenda, Family support, and Rewards. First quartile (<25% in Figure 6) shows fewer features (4 versus 10 in the other quartiles), which are dominated by Family support and Tips and support features, completed by Data record and Personalization.

## Discussion

### Principal Results

We conducted a systematic review to discover the features of the apps that had shown an effect in T1DM management. We found a big gap between research and market in the apps for supporting and empowering T1DM patients. While research is currently testing the effectiveness of mHealth in the improvement of clinical outcomes related to T1DM and therapy adherence, the characteristics of such apps are heterogeneous and not consistently justified. The systematic search discovered 6 studies consisting of mHealth interventions on 266 participants with a study duration ranging from 3 to 12 months. Studies described the app and assessed the user perceived usefulness of the app characteristics. Three of the 6 (50%) studies also reported on user satisfaction. Features of the apps were categorized and merged into a taxonomy of what would be an ideal app for T1DM management and patient empowerment (Figure 2).

The newly proposed taxonomy featured 3 hierarchical levels, the first of which has 10 areas. Subsequent level 2 and level 3 features are embedded into level 1 features, enabling us to detail what type of feature is offered to the app user (eg, Personalization: setting up a user profile). Regarding the interaction of patients and health care professionals, we discovered apps including contact to and support from endocrinologists and diabetes educators (Figure 2). We think that a general practitioner should also be considered as a reference contact point for some cases. Moreover, information about current trends might also be of interest (eg, blood glucose is increasing or decreasing) for patients having a continuous blood glucose monitoring system, or by interpolating self-reported measurements.

Moreover, this study explored systematically the features that are present in apps available at zero cost for users on the internet and mobile apps markets. Following the PRISMA approach, we found 80 apps which were analyzed in detail for 30 days. None of the assessed apps fulfilled the features of the taxonomy of an ideal app, but 1 featured 9 characteristics (BlueLoop). Personalization (70/80, 87.5%) and Data record (64/80, 80.0%) were the 2 top prevalent features, whereas Rewards (3/80, 3.8%) was the less predominant. We did not find an association on the number of features (*P*=.42, F=.81) nor the type of feature (*P*=.20, X^2^_29_=11.7) with the app operative system. Level 2 features were highly heterogeneous, but we highlight the Blood glucose diary featured in 91.4% (64/70) of apps and the low rate of Tailored advice only present in 11.4% (8/70). Level 2 features of Tips and support showed that not all the apps included a user manual (46/60, 76.7%), less than a half (28/60, 46.7%) provided Diet/food information. Only 11.67% (7/60) offered 24/7 medical support specific for diabetes, and 18.3% (11/60) offered Emergency support. To our knowledge, this is the first comprehensive taxonomy specific definition of features and the first review exploring the availability of such features in commercial apps for mHealth interventions in T1DM.

In a secondary analysis apps were classified according to the number of level 1 features and sorted into quartiles (Figure 5). First quartile apps had a regular distribution of the 10 features in the taxonomy, expect Agenda and Rewards. Second and third quartile did not follow this distribution, having a greater frequency of Data record, Personalization, and Data management. The fourth quartile featured Family support and Tips and support.

### Comparison with Prior Work

Patient empowerment is essential for T1DM management and control [19]. Interventions for T1DM with mobile applications are mainly used for managing measurements, reminders and charting data, instead of promoting the self-management of the disease with a comprehensive strategy of skills development. Gamification and social communities have been observed as a key factor for empowering patients, though in our review we confirm these to features as a testimonial.

This research stresses the fact that there is a need to consider the key features to be included in an app for T1DM. This consideration is straightforward related to the ultimate objective of the app. Is it for recording and storing measurements? Is it for managing other aspects as the disposables or the appointments? Is it targeted to empower the user? Is it to build a social community? Goyal and colleagues argued that the design of an app for T1DM young patients had to consider 3 factors: (1) relationship to technology, (2) how this relationship might make a difference to users, and (3) considering when it might not be a suitable mechanism to use [14]. There is a ring to rule them all, but there should not be a T1DM app to rule them all. Patients and relatives will experience an intense evolution since disease onset until blood glucose control. This means that there can be an app for each type of T1DM patient depending on their momentum with the disease and their own context.

The taxonomy of features was designed based on previous clinical interventions [12-17] which have shown a clinical effect on the management of diabetes with mHealth. However, more evidence is needed to correlate specific improvements with the types of features. Many studies revealed the clinical effectiveness of diabetes management based on the definition of the American Association of Diabetes Educators 7 Self-Care Behaviors (AADE7) [20]. The AADE7defines 7 key areas for conducting and effective management of diabetes: (1) monitoring, (2) healthy eating, (3) taking medication, (4) being active, (5) problem solving, (6) healthy coping, and (7) reducing risks. Recently Ye et al [21] reviewed 137 apps for diabetes management based on AADE7 guidelines, concluding that many of the apps were designed for supporting healthy behaviors and a few were for supporting patients at problem-solving, risk reduction, and facing health from a holistic perspective. Our findings support that the current apps are suited for basic management instead of promoting the long-term empowerment of patients.

In 2017, Holtz et al [22] reported the development of a T1DM app with a patient-centered approach. One of the desirable app features that the adolescents mentioned in the evaluation of this study was to be part of a community. Although researchers and clinicians are focusing on intervention based on social networks and mobile apps [23], we do not see such progress when these apps are used for improving clinical endpoints nor in the apps already available to users. Gamification and rewarding are the least prevalent of the features, a reality that should encourage researchers to design new gamification strategies to engage T1DM patients on the importance of self-management.

Gamification and coaching techniques are also a promising feature of mobile health apps for diabetes management. Sannino et al [24] introduced the concept of a constant follow-up of the patient’s performance along with continuous feedback and reward system according to the user behavior and disease control. Although this app was only tested in a pilot set up for assessing usability and satisfaction, some of the features could be introduced in future RCTs to discern which gamification approaches fit better for each type of T1DM user.

The review of apps allowed us to conclude that level 1 features in apps have a balanced distribution (Figure 6). However, rather than focusing on the number of features in an app, research should investigate which of the features contribute to achieving goals effectively. A better understanding of the disease mechanism and treatment effects lead to an improvement of health outcomes [25]. This suggests that small changes in patient environments can have more significant effects on behavior and can be utilized in the self-management of diseases. The mHealth solutions are a promising alternative enabling context-awareness and personalization, but these solutions must be designed integrating evidence-based behavioral change theoretical foundations to be effective [26].

In this study, we have discovered Data record and Personalization as the most prevalent features in mHealth diabetes apps. This finding should be further explored to know how many of these apps that also offer a dashboard for professional management. A recent study has discovered a decrease of the consultation time in type 2 diabetes management by using artificial intelligence and predictive modeling [27,28], and moreover, a review has proved these methods are being progressively established as suitable for use in daily clinical practice [29]. Future research should tap into the application of these methods for supporting both T1DM patients and healthcare professionals on the follow-up and control of this complex disease.

Finally, for the main purpose of disease management apps, rather than investigating the number of app features, we should investigate what kind of features could achieve the goal effectively. In our study, we were not able to distinguish which feature (or combination of features) was helping patients to achieve their goals. Acceptability and usability studies may help to identify the features that have a higher impact on the self-management of the disease, but further research should be conducted to critically identify the sets of features valuable for patients.

### Limitations

A limitation of this study is the set of papers and apps selected for the literature review and the app review. The authors may have omitted significant contributions for both searches due to the incompleteness of the query commands and mismatch in the searches. We have focused our research on apps tested in RCTs with significant clinical end-points. Authors are aware of the relevant research done in the past and conducted in the present in the design of T1DM apps, which do not involve clinical endpoints nor RCTs, which may also contribute to the taxonomy of an ideal app defined in this paper. Another limitation is that the graphical user interface (GUI) of the apps are not evaluated or studied. If the GUI is not appropriate, many features might be less accessible and thus less used.

### Conclusions

This study assessed the existing gap between research and market in mobile health apps for the management of T1DM and the empowerment of patients. The mHealth has potential to support the management of T1DM, to catalyze the information exchange between patients, parents, and caregivers, and to empower and educate patients in the management of T1DM. The current landscape of apps for T1DM does not seem to be close to what researchers promote from RCTs and user-centered design. A majority of the apps mainly support the collection of measurements, and only a few of them offer a wide range of features for a personalize self-management. Rewards and social communities are not yet well adopted in market environments.

## Appendix

Multimedia Appendix 1 Search Strategy and results in PubMed (MEDLINE).

## Abbreviations

AADE7: American Association of Diabetes Educators 7 Self-Care Behaviors
CSV: comma-separated values
FF: Feature Factor
GUI: graphical user interface
HbA _1c_: hemoglobin A_1c_
mHealth: mobile health
RCT: randomized controlled trial
SMBG: self-monitoring blood glucose
T1DM: type 1 diabetes mellitus
