# Supplementary material for: App Features for Type 1 Diabetes Support and Patient Empowerment: Systematic Literature Review and Benchmark Comparison
Source: JMIR Mhealth Uhealth. 2018 Nov 21;6(11):e12237. doi: 10.2196/12237 (PMC6282013; doi:10.2196/12237)
Supplement: Multimedia Appendix 1 [file mhealth_v6i11e12237_app1.pdf]

Appendix A. Search Strategy and results in Pubmed (MEDLINE)

ID	Query	Results
1	diabetes and (mobile phone)	609
2	diabetes AND ((mobile phone) OR (cell phone) OR (smart phone))	873
3	diabetes AND ((mobile phone) OR (cell phone) OR (smart phone) or app)	1262
4	diabetes AND mobile app	506
Including *mobile health* keywords		
5	(Type 1 Diabetes) AND (mobile app)	100
6	(Type 1 Diabetes OR T1DM) AND (mobile app)	103
7	(Type 1 Diabetes OR T1DM) AND (mobile app OR mHealth)	339
8	(Type 1 Diabetes OR T1DM) AND (mobile app OR mHealth OR app)	365
9	((Type 1 Diabetes) OR (T1DM)) AND ((mobile health) OR (app) OR (mobile app) or mobile)))	454
Including *Randomized Controlled Trial* keywords		
10	#9 AND (Randomized Controlled Trial)	79
11	#9 AND (Randomized Controlled Trial OR RCT)	79
12	#9 AND (Randomized Controlled Trial OR RCT OR Randomised Controlled Trial)	79
13	((Type 1 Diabetes) OR (T1DM)) AND ((mobile health) OR (app) OR (mobile app) or mobile) AND (Randomized Controlled Trial OR RCT OR Randomized Trial)	82
Including *self-management* keywords		
14	#1 AND self-management	230
15	#3 AND (self-management or self-care)	303
16	#5 AND (self-management or self-care)	444
17	#9 AND (self-management or self-care)	221
18	#13 AND (self-management or self-care)	48
